# Occupational stress in industry setting in Benin 2019: A cross-sectional study

**DOI:** 10.1371/journal.pone.0269498

**Published:** 2022-06-09

**Authors:** Mênonli Adjobimey, Vikkey Hinson, Rose Mikponhoué, Esdras Hountohotegbe, Elvyre Klikpo, Ibrahim Mama Cissé, Amelée Adjogou, Véronique Dossougbété, Jonathon R. Campbell, Paul Ayélo, Dismand Houinato

**Affiliations:** 1 Doctoral School of Health Sciences of Cotonou, Cotonou, Benin; 2 Occupational Health Department of the National University Hospital of Pneumo-Phtisiology of Cotonou, Cotonou, Benin; 3 Research and Teaching Unit in Occupational and Environmental Health, University of Abomey-Calavi, Cotonou, Benin; 4 Faculty of Health Sciences of Cotonou, University of Abomey-Calavi, Cotonou, Littoral, Benin; 5 Faculty of Health Medicine, University of Parakou, Cotonou, Benin; 6 Louis Pasteur Clinic, Porto–Novo, Cotonou, Benin; 7 Department of Epidemiology, Biostatistics, and Occupational Health, McGill University, Montreal, Canada; University of Palermo, ITALY

## Abstract

**Background:**

Occupational stress is a psychosocial risk in the workplace. Working conditions in industrial settings may lead to occupational stress. In Benin, however, there is little epidemiological data on occupational stress in industrial settings. We aimed to determine the prevalence and factors associated with occupational stress in industrial settings in Benin in 2019.

**Methods:**

This was a prospective, cross-sectional study conducted from January 31 to April 11, 2019, among 15 cotton ginning plants. Sampling was exhaustive for permanent workers and stratified in clusters by shift for occasional cotton gin workers. Data were collected through Karasek and Siegrist questionnaires. Data analysis was performed using R software. Binary multivariable logistic regression was performed. The significance level was p < 0.05.

**Results:**

Of 1883 workers included, 90.8% were male. The median age was 38 years (IQR: 28 years to 49 years). The prevalence of occupational stress was 77.7% (95% CI: 75.8–79.6). Psychological demand was high in 93.0% of workers and 83.9% had low decision latitude. Among the workers, 16.3% had low social support and 89.9% had a low recognition score at work. Factors associated with occupational stress were: being an occasional vs. permanent worker (aOR 6.43, 95% CI 4.18 to 9.88); age less than 38 years (aOR 0.55, 95% CI 0.41 to 0.76); high intensity physical activity at work (aOR 1.33, 95% CI 1.03 to 1.73); working in production vs. administration (aOR 1.59, 95% CI 1.03 to 2.45); spending fewer than 4 years at the current work location (aOR 1.60, 95% CI 1.05 to 2.44); and scoring low for recognition at work (aOR 1.53, 95% CI 1.04 to 2.23). Noise exposure and being a shift worker were significant in univariable analysis, but not multivariable analysis.

**Conclusion:**

Occupational stress is very common among workers in industrial settings. The implementation and evaluation of preventive measures against these risk factors is necessary.

## Introduction

Psychosocial risks, particularly stress, are emerging risks in the workplace. The work environment has a major influence on the mental health and well-being of each employee [1]. This work environment has positive effects when work provides satisfaction and contributes to personal self-fulfillment, or negative effects provoked by situations of stress, inadequate working patterns and schedules, possible situations of abuse and/or harassment [2].

Occupational stress is the reaction that people may have when confronted with work demands and pressures that challenge their coping ability [3]. Recent changes in how work is managed, characterized by tighter control of individual and collective productivity and by attempting to function with minimal manpower, create somatic and cognitive disorders in workers.

Occupational stress has consequences for both workers and companies [4]. Indeed, the conditions of stress at work are capable of creating diseases with more or less serious consequences on the biological, hormonal [5], physical, psychological, and social level of the individual [6,7]. Those psychological consequences are measured through emotional stability [8]. The homeostatic adaptations to stress are regulated by the central nervous system, the neuroendocrine system, and the immune system, which constitute an integrated biological circuit under the control of genes [9]. It has been reported that the work activity, especially that performed during the night, is able to influence the sleep-wake cycle, favoring the development of insomnia. Insomnia would subject the worker to such a stressful condition that it would encourage undesirable behaviors such as the use/abuse of psychotropic substances. A greater propensity of night workers to consume alcoholic beverages than those who work during the day, often in binge-drinking mode, has been reported in the literature [10].

Indeed, inadequate management of occupational stress can lead to a considerable decrease in workers’ performance, burnout [11], an increase in absenteeism from work, and use of psychoactive substances [12–14] like tobacco and alcohol abuse. There is a correlation between occupational stress and alcohol and tobacco abuse [15], especially in rotating or night workers [16,17].

When employee performance declines, it leads to reduced productivity, accidents, and injuries, which result in compensation and treatment costs for injured workers [18–20]. Work-related stress is also a risk for cardiovascular and psychiatric disorders [21,22]. The prevalence of work-related stress varies from one region to another, from one industry to another [3], and according to the tool used to measure stress. In Europe, occupational stress is the second most common work-related health problem, affecting 28% of employees [1]. In most countries, occupational stress has been most studied in the service sector, in particular health care, banking, and transport [23–25]. However, there is a dearth of literature in the industrial sector, which nevertheless may be at high risk of stress.

Cotton ginning sector is one such sector, where, due to market competitiveness in Sub-Saharan Africa, there is great pressure to achieve results. Workers also have other exposures that may cause stress including cotton dust, noise, shift work, and night work [16,26,27]. Faced with these factors, workers may be at risk of occupational stress in the course of their work. Owing to the lack of data on occupational stress, we sought to determine the prevalence and factors associated to occupational stress among cotton gin workers in Benin in 2019.

## Materials and methods

### Study design and setting

A cross-sectional study was conducted from January 31 to April 11, 2019. The study was completed in Benin, West Africa (a low-income country with approximately 12 million inhabitants). Benin has 19 cotton ginning plants, 17 of which are operational. The study was conducted in 15 cotton ginning plants in the country, which included 8 factories in the northern area of the country, 4 in the central area (one factory has two plants), and 2 in the southern area. The two functional plants that were not included in the study were in the northern and central area of the country.

During the cotton season, which lasts an average of 6 months, factories operate 24 hours per day with an 8 hour shift system for most workers. The off-season period is marked by a reduction in production activities and plant staff. There are two sectors in the factory: production and administration. Workers in the production sector are exposed mainly to cotton dust, noise, extended standing, and intense shift work. Workers in the administration sector are exposed to prolonged sitting and work under pressure. Each plant has a functional infirmary staffed by non-occupational health professionals and an Occupational Health and Safety Committee (OHSC). Annual medical check-ups are not routine in all plants. The date of the last medical check-up at the time of the survey varied from 1 to 20 years depending on the plant. The 2019 medical check-up conducted in all ginning plants served as the framework for this study.

### Study population and sampling

The study population consisted of permanent and occasional workers with at least 6 months of service (the equivalent of one cotton season). The inclusion criteria were: age over 18 years, casual or permanent worker status for at least 6 months in the company. The primary exclusion criterion was medical history of psychiatric pathology before beginning work in cotton ginning plants.

There were a total of 229 permanent workers across the plants. The sample size was calculated only for occasional workers assuming a stress prevalence of 50%, an absolute precision of 3.5% at the 95% confidence level, and a design effect of 2. After accounting for 5% refusal or exclusion, the sample size was 1647. The total sample size is therefore 1876. We performed consecutive, exhaustive recruitment for all permanent workers at each plant. For occasional workers, there were four different work-shifts at each plant. We treated each work-shift as a group and did cluster random sampling to select two work-shifts at each plant. We performed consecutive, exhaustive recruitment of occasional workers on these shifts.

### Measurement

The outcome of interest was occupational stress assessed by the Karasek questionnaire [28]. This questionnaire is a measurement scale used as a diagnostic tool to assess the constraints of the psychosocial environment at work. The Karasek questionnaire makes it possible to study three dimensions of the human relationship to work. These are psychological demand (9 items), decision latitude (9 items) and social support (11 items). For each participant, the items were rated from 1 to 4 on the 4-point Likert scale, which made it possible to calculate their individual score.

Decision latitude (DL) evaluates the possibility for each worker to influence their work activity. It covers two dimensions: decisional autonomy (DA) and competence autonomy (CA). The median value of DL that was equal to 70 was used [28]. The score was low when it was less than 70 and high when it was greater than or equal to 70.

Psychological demand (PD) refers to the amount of work to be done, the mental demands and time constraints associated with that work. The median value of PD that was equal to 21 was used. The score was low when it was less than 21 and high when it was greater than or equal to 21.

Karasek’s model permits situating employees on a graph defined according to two axes: decision latitude on the x-axis and psychological demand on the y-axis. This graph is divided by axes corresponding to the median value of each score defining four dials: relaxed, active, stressed, and passive. If a subject had high DL and low PD they were classified as relaxed; if they had high DL and high PD they were classified as active; if they had low DL and high PD they were classified as stressed and if they had low DL and low PD they were classified as passive. Therefore, for our primary outcome of interest, participants scoring <70 on the decision latitude scale and ≥21 on the psychological demand scale were classified as having occupational stress.

Socio-demographic and occupational characteristics (age, sex, marital status, length of employment, details of working conditions as described by the plant manager on type of shift/night work, exposure to noise and chemicals) were collected. Behavioral characteristics defined according to the WHO STEPS questionnaire [29] (smoking, harmful alcohol consumption, and intense physical activity at work) were collected. Information on social support and recognition at work defined according to the Siegrist model were collected [28]. Social support (SS) encompasses all the social and practical interactions from which the worker benefits during their activities, consisting of social support from colleagues (SSC) and social support from the hierarchy (SSH). The median value of SS that was equal to 24 was used. The score was low when it was less than 24 and high when it was greater than or equal to 24. Job recognition or job satisfaction refers to the rewards that employees receive for their efforts at work. This reward includes the status and security of the employee’s job, the respect received, and their salary. The job recognition score was low when it was less than 20 and high when it was greater than or equal to 20.

All data were collected during face-to-face interviews using a digitized smartphone questionnaire with the KoboCollect app. Exact questions and how responses were mapped from the questionnaires to the various outcomes are described in **S1 Methods**.

### Statistical analysis

Data were analyzed with R software (version 4.0.4). Proportions were calculated for categorical variables. Continuous variables were expressed as mean and standard deviation for those with a normal distribution while the median and interquartile range (Q1; Q3) were calculated for non-normally distributed variables. The normality of the distribution was checked with the Shapiro test. We did univariable regression with generalized linear mixed models, treating the business as the cluster, to estimate the association between different factors and occupational stress, expressed as the odds ratio (OR) and 95% confidence interval (95% CI). We did not include variables of alcohol misuse and smoking in regression analyses as these are likely colliders on the causal pathway (i.e., occupational stress and other factors may be associated with alcohol misuse and smoking). Variables in univariable regression with p<0.25 were retained in multivariable regression. We used an alpha of <0.05 to define significance.

### Ethical considerations

Approval by the Ethics Committee of the University of Parakou was obtained (number 0194/CLERB- UP/P/SP/R/SA). Prior approval was obtained from the company’s managers (Number 529/2019/DG/DAF/DAFA/CSRH).

An information note and a consent form were approved by the ethics committee and used during the study. The briefing note is read and explained individually to participants and their expectations are collected before consent is given. Language translations were made for some participants. Written consent was obtained from each participant. The data was collected and processed with respect to confidentiality and human rights.

## Results

### Workers characteristics

**Fig 1:**
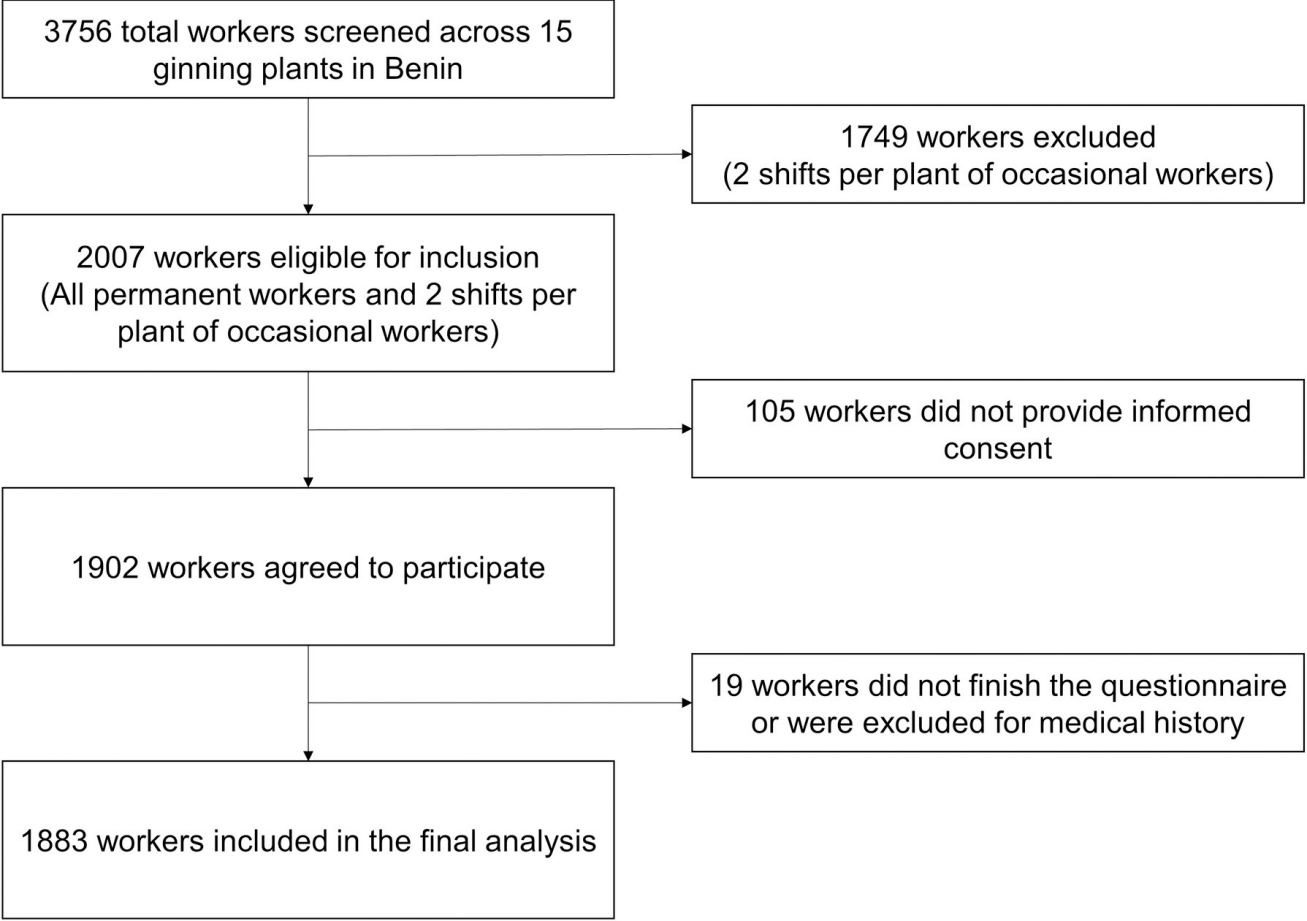
Flow diagram of recruitment of participants at included ginning plants.

The flow diagram of the participants ultimately included in the study is shown in **Fig 1**. A total of 1883 workers were included and 1709 (90.8%) were male (**Table 1**). Their median age was 38 years with an interquartile range (IQR) of 28 to 49 years. The majority of workers were Beninese (99.4%). The majority (82.5%) of participants did not have university-level education and the majority had a partner (76.0%). Few workers had permanent employment status (11.8%) or worked in administrative roles (11.7%). Of included participants, the median (IQR) time at their current role was 3 (2 to 12) years, at the current company was 4 (2 to 12) years, and working in the cotton sector was 5 (2 to 16) years. Most participants were exposed to high levels of noise in the workplace (78.8%). A minority of participants were current smokers or had a history of smoking (14.8%).

**Table 1:** Distribution of cotton mill workers by characteristics (N = 1883).

Location of the factory	Number of employees n (%)
Savalou	122 (6.5%)
Glazoue	121 (6.4%)
Parakou (Factory 1)	124 (6.5%)
Parakou (Factory 2)	136 (7.2%)
N’Dali	129 (6.9%)
Bembereke	144 (7.6%)
Banikoara	141 (7.5%)
Pehunco	122 (6.5%)
Kandi (Factory 1)	144 (7.6%)
Kandi (Factory 2)	143 (7.6%)
Bohicon (Both Plants)	211 (11.2%)
Avogbana	112 (5.9%)
Ketou	115 (6.1%)
Hagoume	119 (6.3%)
**Median (IQR) Age**	38 (28 to 49)
**Sex**	
Male	1709 (90.8%)
Female	174 (9.2%)
**Alcohol and Smoking**	
Alcohol Misuse	1289 (68.5%)
Current Smoker	121 (6.4%)
**Type of Employment**	
Permanent Worker	223 (11.8%)
Occasional Worker	1660 (88.2%)
**Shift worker**	
Yes	1543 (81.9%)
No	340 (18.1%)
**Work Role / Sector of Activity**	
Production	1662 (88.3%)
Administration	221 (11.7%)
**Length of time in position (years)**	
< 3	840 (44.6%)
≥ 3	1043 (55.4%)
**Length of time with the company (years)**	
< 4	822 (43.7%)
≥ 4	1061 (56.3%)
**Length of time in cotton industry (years)**	
< 5	859 (45.6%)
≥ 5	1061 (54.4%)
**Direct exposure to noise**	
Yes	1484 (78.8%)
No	399 (21.2%)
**Exposure to a chemical product**	
Yes	354 (18.8%)
No	1529 (81.2%)

### Prevalence of occupational stress and other outcome measures

**Fig 2:**
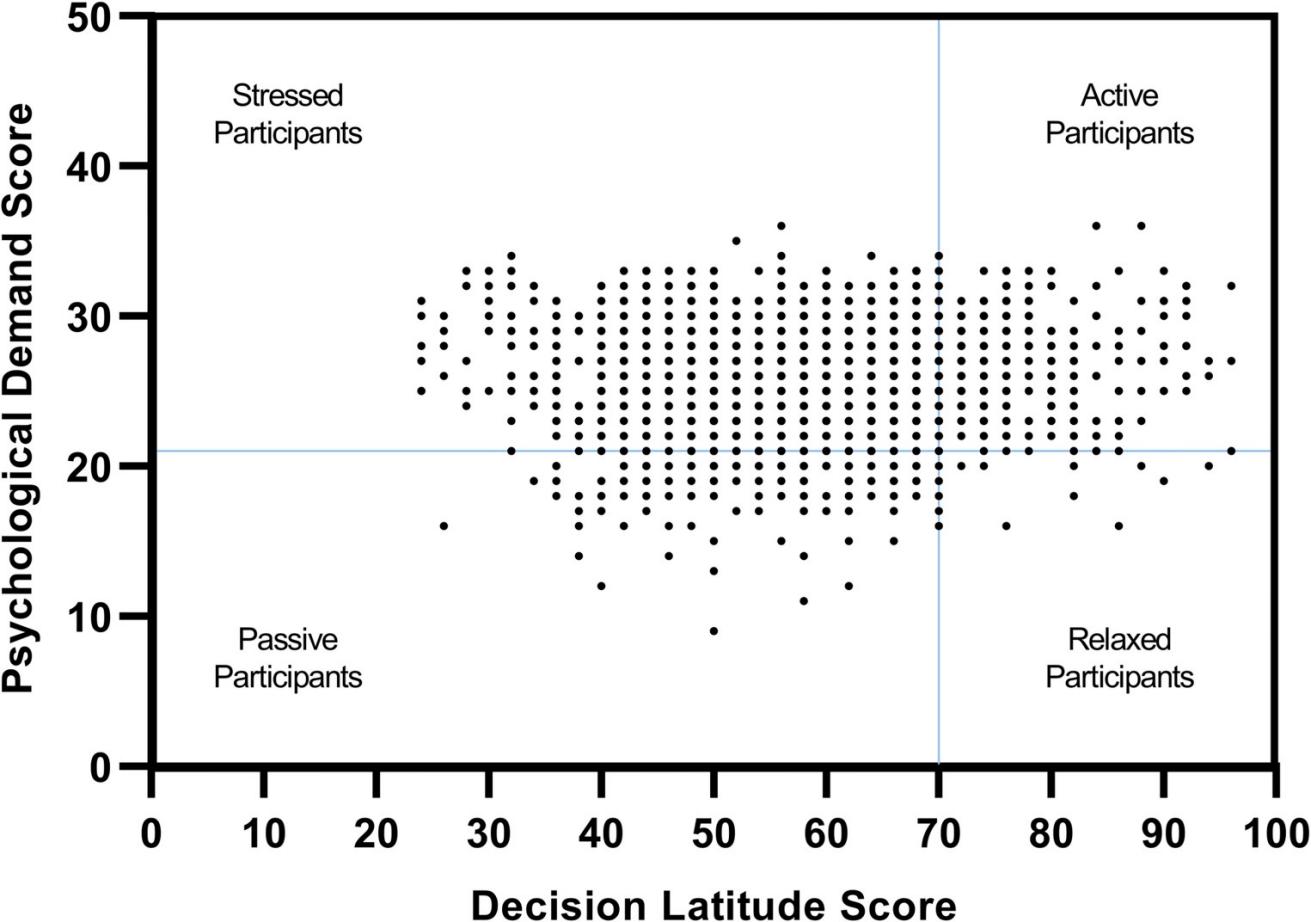
Distribution of workers according to psychosocial constraints using the Karasek dial.

Overall, 1464 of 1883 (77.7%, 95% CI: 75.8% to 79.6%) of workers met the definition for our primary outcome of experiencing occupational stress. The median score for psychological demand was 25 (IQR: 23 to 28) and for decision latitude was 60 (IQR: 52 to 66). Only 131 (7.0%) of participants scored low (<20) on the psychological demand component and 304 (16.1%) scored high (≥70) on the decision latitude component. A large proportion of workers felt they had low recognition at work (1692; 89.9%) Despite these scores, only a small minority of participants reported having low colleague support (13; 0.7%) or having low hierarchical support (37; 2%). **Table 2** shows the prevalence of psychological and organizational constraints by ginning factory and region and **Fig 2** shows the distribution of workers according to the Karasek dial, with psychological demand on the y-axis and decision latitude on the x-axis.

**Table 2:** Distribution of scores for psychological and organizational constraints. All values are N (%).

	N Total	High Psychological Demand	Low Decision Latitude	Low Colleague Support	Low Hierarchical Support	Low Work Recognition
**Total**	1883	1752 (93%)	1579 (83.9%)	13 (0.7%)	37 (2%)	1692 (89.9%)
**By Region**						
Centre	566	521 (92%)	490 (86.6%)	5 (0.9%)	11 (1.9%)	516 (91.2%)
North	1083	1008 (93.1%)	882 (81.4%)	7 (0.6%)	25 (2.3%)	950 (87.7%)
South	234	223 (95.3%)	207 (88.5%)	1 (0.4%)	1 (0.4%)	226 (96.6%)
**By Factory**						
Avogbana	112	106 (94.6%)	98 (87.5%)	0 (0%)	0 (0%)	104 (92.9%)
Banikoara	141	136 (96.5%)	113 (80.1%)	0 (0%)	1 (0.7%)	116 (82.3%)
Bembereke	144	136 (94.4%)	119 (82.6%)	0 (0%)	4 (2.8%)	127 (88.2%)
Bohicon (Two plants)	211	206 (97.6%)	195 (92.4%)	1 (0.5%)	2 (0.9%)	199 (94.3%)
Glazoue	121	114 (94.2%)	97 (80.2%)	1 (0.8%)	0 (0%)	109 (90.1%)
Hagoume	119	114 (95.8%)	108 (90.8%)	1 (0.8%)	0 (0%)	115 (96.6%)
Kandi (Factory 1)	144	133 (92.4%)	120 (83.3%)	1 (0.7%)	0 (0%)	122 (84.7%)
Kandi (Factory 2)	143	129 (90.2%)	121 (84.6%)	1 (0.7%)	4 (2.8%)	129 (90.2%)
Ketou	115	109 (94.8%)	99 (86.1%)	0 (0%)	1 (0.9%)	111 (96.5%)
NDali	129	114 (88.4%)	107 (82.9%)	1 (0.8%)	2 (1.6%)	117 (90.7%)
Parakou (Factory 1)	124	114 (91.9%)	96 (77.4%)	3 (2.4%)	7 (5.6%)	112 (90.3%)
Parakou (Factory 2)	136	126 (92.6%)	110 (80.9%)	1 (0.7%)	4 (2.9%)	123 (90.4%)
Pehunco	122	120 (98.4%)	96 (78.7%)	0 (0%)	3 (2.5%)	104 (85.2%)
Savalou	122	95 (77.9%)	100 (82%)	3 (2.5%)	9 (7.4%)	104 (85.2%)

### Factors associated with occupational stress

In univariable analysis, several factors were associated with occupational stress, however few remained associated after multivariable adjustment (**Table 3**). The strongest factor associated with occupational stress was being an occasional vs. permanent worker, where those who were occasional workers had 6.43 (95% CI 4.18 to 9.88) times higher odds of occupational stress. Age less than 38 years was associated with 0.55 (95% CI 0.41 to 0.76) the odds of occupational stress as compared to persons aged 38 years and older. Other factors associated with occupational stress included high intensity physical activity at work (1.33, 95% CI 1.03 to 1.73), working in production vs. administration (1.59, 95% CI 1.03 to 2.45), spending fewer than 4 years at the current work location (1.60, 95% CI 1.05 to 2.44), and scoring low for recognition at work (1.53, 95% CI 1.04 to 2.23).

**Table 3:** Results of regression analyses for the outcome of occupational stress.

			Univariable Analysis		Multivariable Analysis
	N	N (%) with stress	OR (95% CI)	P value	aOR (95% CI)
**Sex**					
Female	174	137 (78.7%)	1.04 (0.71 to 1.53)	0.838	--
Male	1709	1327 (77.6%)	1.0 (reference)	--	--
**Age (years)**					
< 38	932	749 (80.4%)	1.36 (1.09 to 1.71)	0.008	0.55 (0.41 to 0.76)
> = 38	951	715 (75.2%)	1.0 (reference)	--	1.0 (reference)
**Level of Education**					
Post-Secondary	330	242 (73.3%)	0.74 (0.56 to 0.97)	0.03	1.00 (0.71 to 1.41)
Before Post-Secondary	1553	1222 (78.7%)	1.0 (reference)	--	1.0 (reference)
**Marital situation**					
Lives alone (single)	425	350 (82.4%)	0.69 (0.52 to 0.91)	0.009	0.96 (0.70 to 1.33)
In couple	1458	1114 (76.4%)	1.0 (reference)	--	1.0 (reference)
**High intensity physical activity at work**					
Yes	947	773 (81.6%)	1.75 (1.4 to 2.2)	<0.0001	1.33 (1.03 to 1.73)
No	936	691 (73.8%)	1.0 (reference)	--	1.0 (reference)
**Type of worker**					
Occasional	1660	1377 (83%)	8.4 (6.15 to 11.46)	<0.0001	6.43 (4.18 to 9.88)
Permanent	223	87 (39%)	1.0 (reference)	--	1.0 (reference)
**Work Role/Sector**					
Production	1662	1349 (81.2%)	4.22 (3.12 to 5.71)	<0.0001	1.59 (1.03 to 2.45)
Administration	221	115 (52%)	1.0 (reference)	--	1.0 (reference)
**Seniority at the work position (years)**					
< 3	840	687 (81.8%)	1.54 (1.22 to 1.93)	0.0002	0.87 (0.62 to 1.20)
≥ 3	1043	777 (74.5%)	1.0 (reference)	--	1.0 (reference)
**Seniority in the ginning plant (years)**					
< 4	822	677 (82.4%)	1.7 (1.34 to 2.14)	<0.0001	1.60 (1.05 to 2.44)
≥ 4	1061	787 (74.2%)	1.0 (reference)	--	1.0 (reference)
**Seniority in the cotton sector (years)**					
< 5	859	720 (83.8%)	2.03 (1.6 to 2.57)	<0.0001	1.41 (0.93 to 2.14)
≥ 5	1024	744 (72.7%)	1.0 (reference)	--	1.0 (reference)
**Direct exposure to noise**					
Yes	1484	1186 (79.9%)	1.73 (1.34 to 2.22)	<0.0001	1.22 (0.90 to 1.64)
No	399	278 (69.7%)	1.0 (reference)	--	1.0 (reference)
**Shift worker**					
Yes	1543	1270 (82.3%)	3.55 (2.74 to 4.59)	<0.0001	0.99 (0.66 to 1.49)
No	340	194 (57.1%)	1.0 (reference)	--	1.0 (reference)
**Exposure to a chemical product**					
Yes	354	278 (78.5%)	0.97 (0.72 to 1.3)	0.815	--
No	1529	1186 (77.6%)	1.0 (reference)	--	--
**Recognition at work**					
Low (<20)	1692	1363 (80.6%)	3.56 (2.6 to 4.87)	<0.0001	1.53 (1.04 to 2.23)
High (≥20)	191	101 (52.9%)	1.0 (reference)	--	1.0 (reference)
**Social relationship with hierarchy**					
Low (<8)	37	28 (75.7%)	1.15 (0.31 to 4.3)	0.83	--
High (≥8)	1846	1436 (77.8%)	1.0 (reference)	--	--
**Social relationship with colleagues**					
Low (<8)	13	10 (76.9%)	1.11 (0.51 to 2.41)	0.796	--
High (≥8)	1870	1454 (77.8%)	1.0 (reference)	--	--

OR: Odds ratio; aOR: Adjusted odds ratio.

## Discussion

To our knowledge, this is the first study conducted in Benin to assess occupational stress among cotton ginning plants workers. We found 77.7% of included participants had occupational stress and it was most strongly associated with being an occasional vs. permanent worker. This prevalence of occupational stress is higher than those observed in other sectors of activity. This includes estimates of 27.3% in an insurance company in Benin [30], 54.6% in a port environment in Abidjan, Côte d’Ivoire [31], and 17% in employees across 14 companies in the Tunisian private sector [32]. Our estimates are closer to levels of stress found in the secondary sector of activity, such as a prevalence of 69% among firemen in Senegal [33] and 52% among migrant workers in Cameroon [34].

The strengths of the study lie in the large sample size, the methodology for the selection of participants, national representation of the study, the involvement of all socio-professional categories of the companies, and the use of a validated questionnaire. Indeed, the Karasek questionnaire is the most widely used tool in epidemiological surveys involving a large population, such as the SUMER survey [28,35].

A limitation of our study is that the psychometric qualities of the Karasek questionnaire are more effective in the service sector (interpretation of certain items) [23] whereas our study took place in the industrial sector. The cross-sectional nature of the study is also a limitation. However, the prevention of occupational stress cannot succeed without knowledge of its determinants in each sector, and little data are available in the cotton industry. The variability of the sectors of activity and the types of stress questionnaires used in other studies limits the generalizability of the results.

Being an occasional worker was strongly associated with occupational stress. The occupational status of the worker, especially in an industrial environment, determines the types of tasks that they are assigned. Thus, occasional workers in these ginning enterprises are more subject to tasks of physical strength, requiring rhythmic work where decision latitude is low. In addition, occasional workers are more subject to job insecurity because they generally only work for six months. Several authors have shown the relationship between working conditions, job insecurity and job stress [36–38]. Jigan *et al* found an association between the level of stress and many factors including, the type of work and the professional title, in an oil sector [22].

Age under 38 years appeared to be a protective factor for occupational stress after adjustment for other factors in our study. This may be contrary to expectation as older workers could be more confident in their work and less prone to work-related stress as they age, whereas young workers are still trying to find a work-life balance [22]. However, Go *et al*. showed in nurses that as age increased, work was perceived as more stressful [39]. This difference could be explained by our consideration of the time of workers in their position, company, and sector, which seemed to indicate higher levels of stress for newer workers.

The significant association between the role of the worker (production vs. administration) and occupational stress has also been found in other sectors of activity such as insurance and the oil sector [30,40]. In our study, workers in the production sector had nearly 60% increased odds of being stressed compared to workers in the administration sector. Our results corroborate those of Magroun *et al*. who found a significant association between occupational stress and four sectors of activity: wood production, furniture manufacture, poultry farming and the plastics and rubber industry [32] as well as the findings of Jigan *et al* [40].

High-intensity physical activity at work was significantly associated with occupational stress. Boudet *et al*., after considering the different factors of variation of stress, showed a protective effect of moderate physical activity towards occupational stress [41]. Physical activity is often associated with a reduction in stress and constitutes a method of stress control. However, in the professional context, when the physical workload is intense, it can constitute a risk factor for stress. In fact, in this context, intense physical activity reflects an overload of work or an unsuitability of the work for the person.

Working in the plants for less than 4 years is associated with occupational stress. The association between low job experience and occupational stress was also shown by Jianga *et al*. in oil industry workers [40]. Work experience allows the worker to better manage stressful situations and to better understand the assigned tasks. Workers who have worked for a shorter period of time lack work experience, and therefore need to improve their ability to perform the necessary work tasks. Because of their aspirations for success, the need to perform tedious tasks, and competition among colleagues, they are more likely to experience job stress. This finding is not consistent with the results of Lompo *et al* in the banking sector [42] and Diedhiou *et al* in firefighters [33] which may be attributed to differences between the study subjects and the type of work.

Low recognition at work doubled the risk of job stress. Hinson *et al*. found a similar result and showed that low recognition at work was an additional reason for job strain [30].

In an industrial environment, noise exposure is frequent, and its intensity varies from one workstation to another. Apart from the effects on hearing, some studies indicate that occupational noise exposure may increase the risk of cardiovascular diseases, such as hypertension, coronary heart disease and stroke [43–45]. Some authors, such as Kivimäki *et al*, have concluded that the risk of coronary heart disease increases with occupational stress [21], while Selander *et al* and Eriksson *et al* have shown the synergistic action of noise and occupational stress on cardiovascular risk [46,47]. The results of the present study indicate that occupational noise exposure is associated with occupational stress in univariable analysis but not in multivariable. This could be due to power or suboptimal classification of noise exposure, as it was a classification based on workstation reporting.

Shift work is a frequent form of organization in industrial environments to facilitate the continuity of production. The association found in univariable analysis between shift work and occupational stress has been confirmed by other authors. Indeed, authors have found that occupational stress and shift/night work were associated, both independently and in combination, with an increased risk of poor mental health [22]. Working irregular shifts over a long period of time not only affects the physical health of workers but can also reduce work efficiency and lead to higher absenteeism, which increases the vulnerability of workers. In nurses, there is an association between rotating shift work and increased occupational stress [48]. Among health care workers, the best shifts in terms of reducing the incidence of shift-related disorders are the morning and evening shifts. The night shift is the worst shift in this regard [49]. Therefore, it is suggested that managers make decisions to limit exposure to night work. However, the lack of an association between occupational stress and shift work after adjusting for other factors in our study could be justified by the interactions between the variables.

Consideration of this occupational stress in the improvement of working conditions is necessary. Indeed, the occupational stress component has been integrated into the actions of the OHSC of plants. Thus, an action plan has been set up to map noise, to reinforce the control of the wearing of hearing equipment, to reinforce the personnel entities, and to improve the system of listening to the workers in plants. Several methods for managing work-related stress have been developed around the world: aromatherapy, bibliotherapy, cognitive behavioral therapy, exercise, alternative medicine, mindfulness, stress management, and sensory intervention [50]. Experimentation with some of these techniques in an intervention program is being considered for this population of workers.

## Conclusion

The prevalence of occupational stress among workers in cotton ginning plants is very high. Occasional work, low workplace recognition, and working in production were the main factors associated with stress. Interventions to address or mitigate the impact of these factors on stress should be studied, such as the implementation of stress education, workplace support and recognition programs, and measures to improve job security.

## Supporting information

S1 Methods Description of methods to calculate decision latitude, psychological demand, social support, and job recognition, as well as exact questions asked to participants. (DOCX)
