# Supplementary material for: Occupational stress in industry setting in Benin 2019: A cross-sectional study
Source: PLoS One. 2022 Jun 9;17(6):e0269498. doi: 10.1371/journal.pone.0269498 (PMC9182257; doi:10.1371/journal.pone.0269498)
Supplement: S1 Methods — (DOCX) [file pone.0269498.s001.docx]

**S1. Supplemental Methods**

**Methods of calculating psychosocial risk according to Karasek and Siegrist**

In the descriptions below, “q” refers to the question contained in the modified Karasek-Siegrist questionnaires. The calculations follow those described by Karasek (see Job Content Questionnaire User's Guide) and here: <https://pubmed.ncbi.nlm.nih.gov/9805280/>. The exact questions are at the end of this section.

Decision latitude (DL) evaluates the possibility for each worker to influence his or her work activity; it covers two dimensions, decisional autonomy (DA) and competence autonomy (CA). Thus, DL score = AD score + AC score. AD score = 4 * [q4 + (5-q6) + q8] and AC score = 2 * [q1+ (5-q2) + q3 + q5 + q7 + q9]. In total, DL score = 4 * [q4 + (5-q6) + q8] + 2 * [q1 + (5-q2) + q3 + q5 + q7 + q9]. The French value of the median of DL was used, which is equal to 70. At the end of his evaluation, each worker was classified according to his DL score. The DL is low when the DL score is less than 70 and considered high when the DL score is greater than or equal to 70.

Psychological demand (PD) refers to the amount of work to be done, the mental demands and time constraints associated with that work. PD score = q10 + q11 + q12+ (5-q13) + q14 + q15 + q16+ q17 + q18. The French value of the median PD was used, which is equal to 21. At the end of the evaluation, each worker was classified according to his PD score. PD was considered low when the PD score was below 21 and high when the PD score was greater than or equal to 21.

Social support (SS) encompasses all the social and practical interactions from which the worker benefits during his activities. It consists of social support from colleagues (SSC) and social support from the hierarchy (SSH). SS score = SSC score + SSH score, with SSC score = q23 + q24 + q25+ q26 and SSH score = q19 + q20 + q21 + q22, hence SS = q19 + q20 + q21 + q22+ q23 + q24 + q25+ q26. The French value of the median SS was used, which is equal to 24. Thus, a worker had a low SS if the SS score was below 24 and a high SS if the SS score was greater than or equal to 24. The combination of an occupational stress situation and low social support defines iso strain.

Job recognition or job satisfaction refers to the rewards that employees receive for their efforts at work. This reward includes the status and security of the employee's job, the esteem received and the monetary reward. Questions 27 to 32 of the standardized Karasek-Siegrist stress questionnaire assess this. The job recognition score is given by the formula: (5-q27) + (5-q28) + q29 + q30 + q31 + q32. A job recognition score below 20 is considered low and a score of 20 or more is considered high.

**Questions (in French) – All are scored on a 4 point scale.**

**“fortement en désaccord” = 1**

**“en désaccord” = 2**

**“d’accord” = 3**

**“tout à fait d’accord” = 4**

| 1 | Dans mon travail, je dois apprendre des choses nouvelles |
| --- | --- |
| 2 | Dans mon travail, j’effectue des tâches répétitives |
| 3 | Mon travail me demande d’être créatif |
| 4 | Mon travail me permet souvent de prendre des décisions moi-même |
| 5 | Mon travail demande un haut niveau de compétence |
| 6 | Dans ma tâche, j’ai très peu de liberté pour décider comment je fais mon travail |
| 7 | Dans mon travail, j’ai des activités variées |
| 8 | J’ai la possibilité d’influencer le déroulement de mon travail |
| 9 | J’ai l’occasion de développer mes compétences professionnelles |
| 10 | Mon travail demande de travailler très vite |
| 11 | Mon travail demande de travailler intensément |
| 12 | On me demande d’effectuer une quantité de travail excessive |
| 13 | Je dispose du temps nécessaire pour exécuter mon travail |
| 14 | Je reçois des ordres contradictoires de la part d’autres personnes |
| 15 | Mon travail nécessite de longues périodes de concentration intense |
| 16 | Mes tâches sont souvent interrompues avant d’être achevées, nécessitant de les reprendre plus tard |
| 17 | Mon travail est « très bousculé » |
| 18 | Attendre le travail de collègues ralentit souvent mon propre travail |
| 19 | Mon supérieur se sent concerné par le bien-être de ses subordonnés |
| 20 | Mon supérieur prête attention à ce que je dis |
| 21 | Mon supérieur m’aide à mener ma tâche à bien |
| 22 | Mon supérieur réussit facilement à faire collaborer ses subordonnés |
| 23 | Les collègues avec qui je travaille sont des gens professionnellement compétents |
| 24 | Les collègues avec qui je travaille me manifestent de l’intérêt |
| 25 | Les collègues avec qui je travaille sont amicaux |
| 26 | Les collègues avec qui je travaille m’aident à mener les tâches à bien |
| 27 | On me traite injustement dans mon travail |
| 28 | Ma sécurité d’emploi est menacée |
| 29 | Ma position professionnelle actuelle correspond bien à ma formation |
| 30 | Vu tous mes efforts, je reçois le respect et l’estime que je mérite |
| 31 | Vu tous mes efforts, mes perspectives de promotion sont satisfaisantes |
| 32 | Vu tous mes efforts, mon salaire est satisfaisant |
